# Predictors of a Change and Correlation in Activities of Daily Living after Hip Fracture in Elderly Patients in a Community Hospital in Poland: A Six-Month Prospective Cohort Study

**DOI:** 10.3390/ijerph15010095

**Published:** 2018-01-08

**Authors:** Maria Ganczak, Krzysztof Chrobrowski, Marcin Korzeń

**Affiliations:** 1 Department of Epidemiology and Management, Faculty of Medical Sciences, Pomeranian Medical University, Zolnierska 48, 71-210 Szczecin, Poland; 2 Orthopedic Surgery and Traumatology Ward, Multidisciplinary District Hospital, Dekerta 1, 66-400 Gorzów Wlkp, Poland; krzysztofchrobrowski@me.com; 3 Department of Methods of Artificial Intelligence and Applied Mathematics, Faculty of Computer Science and Information Technology, West Pomeranian University of Technology Szczecin, Zolnierska 46, 71-210 Szczecin, Poland; mkorzen@wi.zut.edu.pl

**Keywords:** hip fracture, surgery, elderly patients, ADL, IADL, risk factors, recovery of function

## Abstract

Objectives: The consequences of hip fractures (HFs) in elderly persons include a deterioration in functional capacity to perform activities that enable independent living. Since prior research into this issue in Central Europe is rather scant, this study sought to assess the change in activities and instrumental activities of daily living (ADL/IADL) after HF surgery among Polish patients, to study predictors of regaining pre-fracture functional status three and six months later, and to evaluate the correlation between ADL and IADL limitations over time. Methods: A prospective study was conducted between 2011 and 2013 in a tertiary hospital in Western Poland. ADL/IADL were evaluated using the Katz index and Lawton scale, respectively. Results: About half (50.8%) of 120 patients (mean age 80.1 ± SD 7.59) had cognitive impairment (CI). Patients with CI were older (*p* = 0.002) and had lower scores for pre-fracture ADL/IADL (*p* = 0.001 and *p* < 0.001, respectively). Six months after HF, 33.3% of patients failed to return to their pre-fracture ADL and 62.5% failed to return to pre-fracture IADL; 20% of those who could walk before HF were unable to walk after six months. The pre-fracture Spearman correlation coefficient between ADL and IADL summary scores was 0.46; it increased to 0.70 at three months after HF surgery and 0.77 at six months (*p* < 0.0001). Regaining ADL after six months was more likely in patients with pre-fracture intact intellectual function and independence in pre-fracture ADL; regaining IADL, in younger patients and those with higher pre-fracture IADL scores. Conclusions: Impairment in functional performance is common after HF surgery. ADL and IADL were strongly correlated in these patients, with this increasing over time. Functional outcomes after HF were more dependent on patient characteristics than treatment-related factors. Therefore, more emphasis should be directed towards the pre-fracture period and, in particular, maintaining cognitive function and preserving functional capacity in older persons at high risk of HF.

## 1. Introduction

Geriatric hip fracture (HF) is one of the most frequent and severe complications of a fall and remains an important social, medical, and economic problem worldwide due to the ageing population and increasing life expectancy [1,2,3]. The incidence of HFs varies by age, sex, anatomical site of fracture, race and ethnicity, geographical region, and season [2,4]. About 75% of HFs occur in women, due to osteoporosis following menopause. Almost half of HFs is reported in patients aged 80 years or older [2,5]. Overall, the lifetime risk of HF is 16% for Caucasian women and 5% for men; after the age of 50, this increases to 23% and 11% respectively [5].

In 2000, an estimated 1.6 million HFs occurred worldwide, and this number is expected to increase to 6.3 million by 2050 [2,4,5]. Numerous studies have examined HF rates in different regions of the world; greater than 10-fold differences have been found [5]. HFs are more prevalent in Europe and the USA. In Europe, the highest HF incidence rates are reported in Scandinavia, while high rates are also observed in other Western European countries (Belgium, Germany, Switzerland, and Austria) and Central Europe (Hungary, Czech Republic, and Slovakia). These countries also account for a major proportion of the HF research, while there is a corresponding lack of data for some other countries, such as Poland. Polish data on HF incidence are not only scant but also outdated [4]. The incidence is estimated at 283/100,000, similar to rates observed in Germany, but almost five-fold lower than in Sweden [4,5].

Most HF patients experience balance impairments, decreased muscle strength, and loss of functional capacity [1,2,4,5]. The latter is specifically related to activities of daily living (ADL) [3]. ADL can be divided into basic and instrumental (IADL) activities. Whereas basic ADLs diminish in the late-middle and later phases of the illness, IADLs diminish earlier due to the fact that their performance requires mental as well as physical capacity [6].

Only 50–71% of HF survivors are likely to regain their pre-fracture levels of mobility 12 months after HF, and 10–20% will be institutionalized permanently [3,6,7]. Data from the HF patients showed that (after adjusting for age, pre-existing chronic medical conditions, and disabilities) two years after a HF, they were more likely to be community immobile and functionally dependent compared to controls [3,8]. A recent study shows that 5964 Disability Adjusted Life Years (DALYs) (27 per 1000 individuals) were lost due to HFs, 1230 (20.6%) of which were in the 75–79-year-old group; 4150 (69.6%) DALYs were attributed to disability [9]. Additionally, HFs seem to reduce the patient’s remaining life expectancy; excess mortality rates in the first year after a fracture are as high as 12–35% [1].

Many studies have identified specific demographic or health pre-operative indicators, as well as operative and post-operative predictors of long-term functional outcome among older HF patients who underwent surgical treatment. Age, gender, pre-fracture functioning, health status, including cognitive status, and fracture type are the most common patient-related factors [3,10,11,12,13,14].

Cognitive impairment (CI) is a predictor of functional outcomes among older HF patients and is also related to an increased risk of fracture [15]. The prevalence of CI is believed to be three to six times higher for hospitalized HF patients than other patients [15]. Patients with different levels of CI may present diverse demographic characteristics on admission and may also perform differently regarding operative and post-operative factors, such as time to surgery, type of anesthesia, type and surgical treatment duration, time to mobilization, length of hospital stay, and post-discharge rehabilitation. However, it should be noted that studies which evaluated this issue are rather scant.

Furthermore, although several studies assessed predictors for a change in ADLs and IADLs after HF in elderly patients [3,10,11,12,13,14], the possible correlation between limitations in both types of activities with the passage of time have yet to be fully evaluated. Knowledge of how mobility limitations after surgery and prolonged physical disability influence both functionalities at the same follow-up time is needed to gain better understanding of deficits and resources of the older patients. This, in turn, may allow better assistance and support during the rehabilitation process.

## 2. Objectives

To gain better insights into the consequences of HF from the perspective of a Central European country, we aimed to assess the changes in ADL and IADL attributable to HF at three and six months after surgery, determine which pre/intra/postoperative factors predict functional ability post-surgery, and evaluate a correlation between ADLs and IADLs over time. To our knowledge, similar studies have yet to be carried out in our region.

## 3. Materials and Methods

### 3.1. Design, Setting, Population, and Sampling

**Figure 1:**
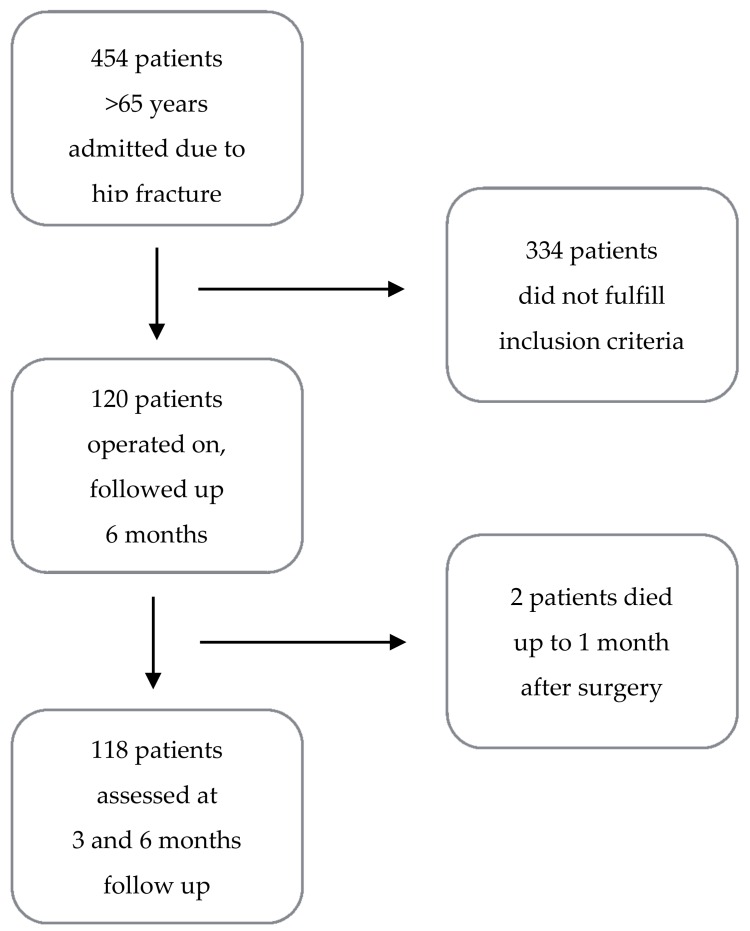
Recruitment and follow-up process flow chart.

A prospective single-center observational study was performed on consecutive patients who presented with HF at the orthopedic ward of a tertiary 800-bed hospital in Gorzow, in western Poland, between December 2011 and November 2013. Inclusion criteria were as follows: >65 years of age, a femoral (neck, inter-trochanteric, or sub-trochanteric) HF, and informed written consent to participate in the study. During the study period, there were 454 HF cases. Patients with poor cognitive status (0–2 points on the *Short Portable Mental Status Questionnaire*—SPMSQ [16]), treated conservatively, and with fractures associated with a high-energy injury or poly-trauma were excluded. The application of these criteria left 120 patients available for follow-up (Figure 1). The study was approved by the local ethical committee (Komisja Bioetyczna przy Okręgowej Izbie Lekarskiej w Gorzowie Wlkp., KB OIL 1/2011; date of approval: 28 December 2011).

### 3.2. Data Collection

All patients underwent an interview by one of the authors (Krzysztof Chrobrowski) and a trained physiotherapist to collect their socio-demographic data (age, gender, and residency), and medical history (cognitive status, co-morbidities). Before the completion of the questionnaire, written consent was obtained from every patient. For cognitively impaired patients who were unable to answer the entire questionnaire, a proxy was asked to respond to the questions. Such proxy responses accounted for 3.3% of the interviews conducted. Cognitive status was assessed with the SPMSQ. The SPMSQ has a maximum score of 10, and any score greater than or equal to 8 indicated normal cognition, 6–7 points indicated mild CI, and 3–5 points indicated moderate CI [16]. Co-morbidities were assessed through the *Charlson Comorbidity Index* (CCI) [17]. Each co-morbidity category had an associated score (from 1 to 6), based on the adjusted risk of mortality or resource use, and the sum of all the scores resulted in a single co-morbidity number for each patient, with 0 indicating no co-morbidity. The higher the score, the more likely the predicted outcome would result in mortality or higher resource use [18].

Pre-fracture functional status was assessed using the Katz ADL index and Lawton-Brody IADL scale; this assessment was repeated at three and six months after surgery. The ADL index [19] measures the patient’s ability to independently perform six activities: transferring, bathing/showering, dressing, self-feeding, toileting, and continence; a score of 6 indicates full function. The IADL Lawton-Brody scale [20] measures the functional impact of emotional, cognitive, and physical impairments. The scale assesses eight activities: use of the telephone, food preparation, mode of transportation, laundry, shopping, housekeeping, responsibility for own medications, and ability to handle finances. Each activity is scored on a three-point scale: independent (3 points), some help needed (2 points), and dependent (1 point); a score of 24 indicates full function. In addition, pre-operative ASA (The American Society of Anesthesiologists) class [21], time to surgery, type of anesthesia, type and duration of surgical treatment, time to mobilization, possible complications, and length of hospital stay were recorded at discharge.

Standard radiographs were used to classify all fractures. The pattern of the fracture was defined by the use of the *Arbeitsgemeinschaft für Osteosynthesefragen*—AO (intertrochanteric HFs) and AO/Garden (neck HFs) classification systems. Criteria for surgical decisions were based on standardized department protocols. Depending on the age and fracture pattern, patients with femoral neck fractures were treated by hemiarthroplasty (for older and low-activity patients with displaced fractures), total hip arthroplasty (for older, more active patients or patients with pre-existing arthritis), or closed reduction and internal fixation (non-displaced, stable neck fracture). Those with intertrochanteric or sub-trochanteric fractures were mainly treated by closed/open reduction and internal fixation.

The day after surgery, patients were asked to take a sitting position in bed and received physiotherapy until discharge. Two days after surgery, full weight bearing on the operated limb was permitted except in cases with medical contraindications for assuming the standing position or concerns about the stability of the implant or fracture. At discharge, patients were referred for rehabilitation. Discharge options included the hospital ward, a rehabilitation facility in their community, or home. To obtain follow-up data, telephone interviews were performed by one of the study investigators (Krzysztof Chrobrowski) at three and six months after HF. Interview records were used to collect follow-up data from patients (in some cases this came from relatives or caregivers) to assess their functional status.

### 3.3. Statistical Analysis

Data analysis was carried out using STATISTICA PL, Version 12.5 (StatSoft, Kraków, Poland) and R software (R Foundation for Statistical Computing, Vienna, Austria) [22]. Categorical variables were expressed as frequency and percentages, and continuous variables were reported as mean ± standard deviation. Basic, pre-fracture functional status (scoring) was evaluated for every participant by the use of the Katz ADL index and Lawton-Brody IADL scale to assess if each patient had regained the pre-fracture score three and six months after surgery. These were primary outcome variables and we aimed to identify explanatory variables significantly associated with these outcomes. For all outcomes, before constructing multivariate analysis models, a bivariate analysis was performed. Bivariate analysis assessed demographic/patient characteristics: age (<80/≥80 years), gender, residence (urban/rural), cognitive status (intact intellectual functioning/mild–moderate intellectual impairment), CCI index (score), ASA class (1–2/3–4); as well as operative factors: type of fracture (trochanteric/femoral neck), time to surgery (<48/≥48 h), type of anesthesia (local/general), type (osteosynthesis/arthroplasty) and surgical treatment duration (minutes); and post-operative factors: time to mobilization (≤2/>2 days), length of hospital stay (≤14/>14 days), and post-discharge rehabilitation (yes/no). Categorical variables groups were compared using the χ^2^ test with Yates correction and Fisher’s exact test; the Mann-Whitney test and the two-sample *t*-test or Kruskal-Wallis test were used for numeric variables. Kendall Tau correlation was used to assess correlation between ordinal variables. *p*-values < 0.05 were considered statistically significant. For the predicted outcome variables listed above, standard single-outcome logistic regression models were built; all models were reduced by the use of a stepwise selection [23]. Unstandardized regression coefficients in the regression model were used to assess any change in the model. A change in coefficients was compared and used to determine any variable change. For binary data, the exponent of the coefficient is interpreted as the odds ratio (OR) [24]. Spearman’s rank correlation coefficient was used to measure the correlation between the ADL scoring with IADL scoring.

## 4. Results

### 4.1. Baseline Characteristics and Surgical Features of Patients

A flow chart of the recruitment and follow-up process is presented in Figure 1. Non-respondents (*n* = 334) were found to be significantly younger (*p* = 0.003) than respondents. No differences were found regarding gender (*p* = 0.45) and residence (*p* = 0.69) between the groups.

Baseline participant characteristics by SPMSQ score are presented in Table 1. The mean age of the study population was 80.1 (±SD 7.59) years, 60.8% being over 80 at the time of the study. Of the 120 patients who were assessed, 81.7% were female; 61.7% lived in urban areas.

About half of the patients (50.8%) had CI (scored less than 8 points on the SPMSQ): 26.6% were moderately impaired, 24.2% were mildly impaired, and 49.2% were cognitively intact. All patients were considered to have co-morbidity, and 50.0% presented a high co-morbidity index (scored 4 or more on the CCI). About two-thirds of the patients (60.9%) were classified in the ASA-3 or 4 categories.

In terms of fracture type, 62.5% of the fractures were intertrochanteric. More than half of the patients (51.7%) were operated on up to 48 h after admission. Perineural (block) anesthesia was performed in most surgeries (89.2%). Fractured bone osteosynthesis was performed on nearly two thirds of the patients (65.8%), arthroplasty on 34.2%, and one-fourth (25.8%) needed a blood transfusion. Mean surgery time was 59.4 min (SD ± 20.1) while mean hospital stay was 14.1 days (SD ± 7.7). Mean time between surgery and mobilization was 2.0 days (SD ± 1.6).

Patients with an SPMSQ score ≥8 were younger and had lower CCI scores and ASA class. They also had higher scores for pre-fracture IADL (Table 2). The rate of pre-fracture functional independence in basic ADL (i.e., ADL Index 5–6) was significantly higher in the cognitively intact group and the group with mild CI compared to the group with moderate CI while the rate of pre-fracture severe disability (i.e., ADL score 0–2) was significantly higher in the latter group (*p* < 0.001 for all comparisons). The rate of pre-fracture functional independence in IADL (i.e., IADL score 19–24) was significantly higher in the cognitively intact group compared to the other groups (*p* < 0.001). No differences were found in terms of gender, place of residence, type of fracture, time interval between admission and surgery, mean surgery time, type of anesthesia, type of procedure, blood transfusion, length of stay, and time from surgery to mobilization among the three SPMSQ groups.

During their hospital stay, six patients (5.0%) developed complications; two patients died within one month of surgery, yielding a mortality rate of 1.7%.

All patients received physiotherapy up to discharge. After discharge, 32 (26.7%) patients were referred to a rehabilitation ward for a period of three weeks, nine (7.5%) received other forms of physio-therapy after discharge (3.3% practiced home rehabilitation and 4.2% underwent rehabilitation at an inpatient facility), whereas 79 (65.8%) did not receive any form of rehabilitation after discharge.

### 4.2. Change in Functional Status

The mean pre-fracture, three- and six-month ADL index was 5.7 (SD ± 0.8), 5.0 (SD ± 1.6), and 4.8, (SD ± 1.9), respectively. In comparison to pre-fracture status, a deterioration in ADL scoring was significant at both follow-up intervals (*p* < 0.001 and *p* = 0.004, respectively). Regarding IADL, the mean pre-fracture, three- and six-month index was 18.5 (SD ± 5.0), 15.0 (SD ± 5.5), and 16.0 (SD ± 6.1), respectively. A worsening in IADL scoring six months after HF surgery was observed in comparison to pre-fracture status (*p* < 0.001).

Three months after HF surgery, 85 (70.8%) patients regained ADL function at the pre-fracture level, and six months after surgery, the number decreased to 80 (66.7%). Changes in ADL as assessed by χ^2^ for trend (0–3–6 months) were significant (*p* < 0.0001). All patients were able to walk before HF; three months after surgery, 16 patients (13.3%) lost the ability to walk, which this increased to 20.0% (*n* = 24) three months later. Regarding IADL, 19 (15.8%) patients regained their pre-fracture level three months after surgery, and this increased to 37.5% (*n* = 45) at six months; *p* < 0.0001.

### 4.3. Prognostic Factors for Functional Status at Follow-Up

Predictors of regaining a pre-fracture ADL index score are reported in Table 2. When multi-variate logistic regression was used to assess these determinants at three months after HF surgery, females (OR = 5.79, 95% CI: 1.35–24.88; *p* < 0.05) and patients with intact intellectual functioning (OR = 11.67, 95% CI: 3.13–23.54; *p* < 0.001) were more likely to regain the pre-fracture ADL. At six months, a return to pre-fracture ADLs was related to intact intellectual functioning (OR = 7.20, 95% CI: 2.19–22.84; *p* = 0.001) and pre-fracture ADL index (OR = 1.23, 95% CI: 1.02–3.17; *p* < 0.001).

Intact intellectual functioning (OR = 7.19, 95% CI: 1.09–27.40; *p* < 0.05) and high pre-fracture IADL index (OR = 4.0, 95% CI: 1.62–9.73; *p* < 0.01) were predictors of return to a pre-fracture IADL score at three months after HF surgery (Table 2). The only determinants of return to pre-fracture IADLs at six months were younger age (OR = 4.11, 95% CI: 1.47–11.48; *p* < 0.01) and pre-fracture IADL index (OR = 1.17, 95% CI: 1.03–1.40; *p* < 0.05).

### 4.4. Correlation between ADL and IADL

**Figure 2:**
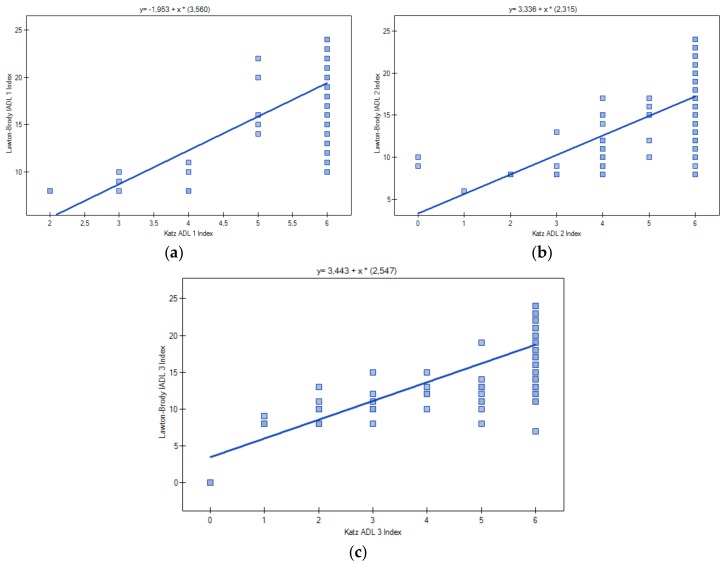
Spearman correlation coefficient of sum scores of ADL and IADL: pre-fracture (**a**); three, and six months after HF surgery (**b**,**c**).

A positive correlation was observed between the ADL index at baseline, three months, and six months after surgery (*p* < 0.001; Spearman’s R > 0.45). A similar correlation was observed for IADL scores (*p* < 0.001; R > 0.45). Additionally, a correlation between the summary ADL and IADL scores was found. The pre-fracture Spearman correlation coefficient was 0.46, and was 0.70 at three months and 0.77 (*p* < 0.001) at six months (Figure 2).

## 5. Discussion

### 5.1. Results Overview

Six months after surgery for HF, two-thirds of our patients regained their pre-fracture ADL levels, and one-third regained their IADL levels. The pre-fracture status was the most important determinant of function at follow-up. Poor cognitive function was associated with worse ADL while older age was associated with worse IADL. In addition, a strong, increasing correlation was found between ADL and IADL scores.

### 5.2. Change in Functional Status

The proportions fully recovering post-surgery in our study were consistent with previous research [7,13,14,25]. According to a review by Dyer et al. [7], about 30% of HF patients did not regain their pre-fracture level of independence in ADL. Moreover, ADL recovered most rapidly during the first three months, with only minor improvements subsequently. This finding is in line with a study conducted in Taiwan [25]. However, studies in the U.S. [26] and Canada [27] have found significant improvements in ADL between three and six months. The difference may be due to greater accessibility of rehabilitation services in those countries, as only one-third of patients in our study received any form of rehabilitation up to six months after HF surgery. Yet the health systems are likely to differ between those countries and Poland, which therefore makes it difficult to make detailed comparisons.

Our patients had more difficulty regaining function in IADL than in ADL. This might have been due to the different models of regeneration of both functionalities and the sensitivity of these regarding factors related to operative treatment and rehabilitation, as similarly reported in previous studies [25,28]. Other studies have confirmed that about half or fewer individuals experiencing HF regain their pre-fracture level of independence in IADL [7,25,28]. In general, studies in the US suggest that IADL limitations were more prevalent among the elderly than ADL limitations [7,26].

### 5.3. Correlation between ADL and IADL

A strong correlation between ADL and IADL scores observed in our study (0.70 at three months and 0.77 at six months), was noticed by other researchers [29,30]. Roehrig et al. [29] found a correlation of 0.65 in elderly cancer patients, while an Iranian study of war veterans found a correlation 0.58 [30]. Both types of limitations tend to increase with age [31]. Furthermore, limitations in ADL (transferring, dressing, self-feeding, toileting, and continence) may cause difficulties with IADL (food preparation, laundry, house-keeping, responsibility for own medications, transportation, shopping, and ability to handle finances). This may explain why, in our study, the correlation became stronger following a severe trauma from HF and the associated mobility limitation after surgery. However, the direction of this causal relationship may not be obvious. In a Norwegian study among older HF patients, pre-fracture IADL was a strong predictor of dependency in ADL [32].

### 5.4. Predictors for Functional Outcomes: ADL and IADL

Age and gender were significant factors in recovery after HF in our study. Younger age was a strong predictor of regaining IADLs at the second follow-up. Patients less than 80 years old were four times more likely to regain pre-fracture IADLs when compared with older participants. As noted by others, older patients may be less able to regain the skills required for daily living, such as using the telephone or food preparation, than their younger counterparts [3,10,11,26]. Females had five times greater odds of regaining their pre-fracture ADL level compared with males three months after HF surgery; however, the association was not observed three months later. Previous studies of the effect of gender on ADL recovery have been inconsistent [32,33,34], suggesting that further research of this issue is needed.

Half of our study population had CI; it was more likely to be found in older patients. This is not surprising, as the associations of CI with older age and HF were previously reported [35,36]. A comparison of proportions of CI patients reported in our study with other studies which analyzed baseline cognitive status in HF patients was difficult due to the fact that patients with severe CI were excluded from the present survey. CI was seen in about 50% of older hospitalized HF patients from Italy and Serbia [15,37], and in 69% of Polish geriatric patients [38]; 7–11% of patients had severe impairment. The high percentage of CI in HF patients represents a major challenge, as cognitive disability is related to increased risk of adverse postoperative outcomes, functional decline, and mortality [15]. Introducing systematic, proactive identification of CI patients in a preoperative check, staff training sessions, as well as interdisciplinary treatment might better help in regaining pre-fracture level of independence and in protecting mortality in this group [15,38].

The study provides further evidence of the role of cognitive function in regaining ADL and IADL abilities after HF surgery. Cognitively intact and mildly impaired patients had a significantly higher pre-fracture ADL index and higher scores for pre-fracture IADLs in comparison to those moderately impaired, which is in line with previous findings [15,39]. Furthermore, six months after HF surgery, those with higher SPMSQ scores (8–10) had seven-fold higher odds to regain the pre-fracture ADLs compared with CI patients. Poor cognition has been previously identified as a strong predictor of functional limitations in such patients [7,12,14,32,39]. The mechanism may involve a tendency among patients with CI to be less actively engaged in rehabilitation and less motivated to return to previous activities [11]. According to Chaves et al. [40] impairments in functional status may be secondary to cognitive problems already present in elderly individuals.

Pre-fracture status was another predictor of both ADL and IADL outcomes post-surgery. This is in line with a number of studies which describe the role of past functional level as a facilitator of recovery [3,11,12,14,24,32]. It may also indicate that patients who are more physically active before fracture may have less difficulty regaining their function. On the other hand, factors such as ASA index, fracture pattern, the specific surgical treatment, length of stay, and post-discharge rehabilitation were not predictive of long-term functional outcomes among HF patients. Similar results were reported by others [3,11].

The above observations call for a reconsideration of the current focus on pre- and post-operative indicators in HF and suggest that shifting attention to the pre-fracture period may be warranted. Surgical outcomes may be improved by interventions aimed at improving mental and physical health of older persons at increased risk of fracture, enhancing their bone quality, reducing the risk of falls, and implementing integrated geriatric care. In a Polish context, this may be a challenge, as access to geriatric care in Poland is worse than in many other European Union countries [41]. Geriatric care within the National Health System receives relatively modest funding and has not been recognized as a priority by politicians and decision-makers.

## 6. Limitations

This study has several limitations. Firstly, only about one in four patients (26.4%) hospitalized at the orthopedic ward at the time of the study fulfilled inclusion criteria. However, no differences regarding basic demographic characteristics were found between groups, except age. Secondly, baseline data were collected retrospectively which may introduce a recall bias. The fact that time between an injury and data collection was short and we used standardized questionnaires constructed in order to maximize accuracy and completeness might reduce the recall effect. The follow-up period (six months) was not long, however, literature review shows that most activities are recovered during the first four to six months after HF surgery and that only minor further improvement occurs thereafter [15,42]. The sample was drawn from one tertiary hospital, which limits generalizability outside of the studied population. Finally, the CIs for some selected variables are wide, indicating that the sample size was relatively small. Therefore, the study needs to be conducted at a national level whereby future results may have more generalizability.

## 7. Conclusions

The present study assessed changes in functional status and key elements affecting the functional prognosis of patients from a Polish tertiary hospital who underwent HF surgery. Our results mostly support previous findings from other countries, both in the European region and outside Europe [7]. Functional impairments are common in these patients. Functional outcomes are patient-dependent rather than institution- or medical-care-dependent, with previous functional and cognitive status acting as the most important elements in regaining pre-fracture parameters. As such, these factors should be of special interest to clinical staff in planning individualized treatment and rehabilitation. The study shows that comprehensive geriatric care, which helps to maintain good physical and mental fitness in the elderly population, might have a beneficial influence on recovery after HF.

## Figures and Tables

**Table 1:** Participant characteristics by baseline Short Portable Mental Status Questionnaire (SPMSQ) score.

Variable	Total	SPMSQ 8–10	SPMSQ 6–7	SPMSQ 3–5	*p*
	*N* = 120	*N* = 59	*N* = 29	*N* = 32	
*Age* (years)					
Mean	80.1	79.6	83.3	82	0.002 *
*Gender*					
Female	98 (81.7)	47 (79.7)	24 (82.8)	27 (84.3)	0.84 *
Male	22 (18.3)	12 (20.3)	5 (17.2)	5 (15.7)	
*Residence*					
Urban	74 (61.7)	37 (62.7)	20 (69.0)	16 (50.0)	0.29 *
Rural	46 (38.3)	22 (37.2)	9 (31.0)	16 (50.0)	
*CCI Index*					
1–3	60 (50.0)	41 (69.5)	13 (44.8)	6 (18.8)	<0.001 **
4–7	60 (50.0)	18 (30.5)	16 (55.2)	26 (81.2)	Tau −0.43
*ADL index*					
5–6	110 (91.7)	58 (98.3)	29 (100.0)	23 (71.9)	<0.001 **
3–4	8 (6.7)	1 (1.7)	0 (0.0)	7 (21.9)	Tau 0.35
0–2	2 (1.6)	0 (0.0)	0 (0.0)	2 (6.2)	
*IADL score*					
19–24	65 (54.2)	54 (91.5)	10 (34.5)	1 (3.1)	<0.001 **
13–18	36 (30.0)	4 (6.8)	19 (65.5)	13 (40.6)	Tau 0.77
9–12	13 (10.8)	1 (1.7)	0 (0.0)	12 (37.5)	
≤8	6 (5.0)	0 (0.0)	0 (0.0)	6 (18.8)	
*Type of fracture*					
Intertrochanteric	75 (62.5)	38 (64.4)	18 (62.1)	20 (62.5)	0.97 *
Femoral neck	44 (37.5)	21(35.6)	11 (37.9)	12 (37.5)	
*Time to surgery*					
≤48 h	72 (51.7)	21 (35.6)	15 (51.7)	12 (37.5)	0.32 *
>48 h	48 (48.3)	38 (64.4)	14 (48.3)	20 (62.5)	
*Type of anesthesia*					
Local	107 (89.2)	55 (93.2)	26 (89.7)	26 (81.3)	0.20 *
General	13 (10.8)	4 (6.8)	3 (10.3)	6 (18.7)	
*ASA class*					
1–2	47 (39.1)	31 (52.5)	10 (34.5)	6 (18.7)	0.001 **
3–4	73 (60.9)	28 (47.5)	19 (65.5)	26 (81.3)	Tau 0.27
*Type of procedure*					
Osteosynthesis	79 (65.8)	42 (71.2)	18 (62.1)	19 (59.4)	0.46 *
Arthroplasty	41 (34.2)	17 (28.8)	11 (37.9)	13 (40.6)	
*Blood transfusion*					
Yes	31 (25.8)	10 (16.9)	9 (31.0)	12 (37.5)	0.07 *
No	89 (74.2)	49 (80.1)	20 (69.0)	20 (62.5)	
*Mean length of surgery* (min)					
59.4		58.2	62.9	61.6	0.49 **
*Mean length of hospital stay* (days)					
14.1		15.2	13.2	12.8	0.54 **
*Mean time from surgery to mobilization* (days)					
2		2.1	2.6	2.4	0.54 **

* χ^2^ test; ** Kendall tau-b correlation.

**Table 2:** Logistic regression model: association of ADL index and IADL score, three and six months after HF with selected variables (OR’s estimates, significance of coefficient using Wald’s test); Poland 2011–2013, *n* = 120.

Variable	ADL				IADL			
	3 Months		6 Months		3 Months		6 Months	
	OR	95% CI	OR	95% CI	OR	95% CI	OR	95% CI
*Gender*								
Male	1		1		1		1	
Female	5.79	1.35–24.88 *	2.11	0.55–8.04	1.96	0.36–10.62	1.03	0.35–3.06
*Age*								
≥80 years	1		1		1		1	
<80 years	2.23	0.68–7.05	1.23	0.40–1.24	1.11	0.31–3.97	4.11	1.47–11.48 **
*SPMSQ index:*								
Cognitive impairment	1		1		1		1	
Intact intellectual functioning	11.67	3.13–23.54 ***	7.2	2.19–22.84 ***	7.19	1.09–27.40 *	1.2	0.48–3.13
*Pre-fracture ADL index*	1.13	0.63–2.02	1.23	1.02–3.17 *				
*Pre-fracture IADL score*	-	-	-	-	4	1.62–9.73 **	1.17	1.03–1.40 *
R^2^	0.376		0.374		0.189		0.165	

ADL: activities of daily living; IADL, instrumental activities of daily living; HF, hip fracture; OR: Odds Ratio; 95% CI: 95% Confidence Intervals; R^2^: Coefficient of determination; Statistically significant at * *p* < 0.05, ** *p* < 0.01, *** *p* ≤ 0.001.
